# Salt Stress Effects on Secondary Metabolites of Cotton in Relation to Gene Expression Responsible for Aphid Development

**DOI:** 10.1371/journal.pone.0129541

**Published:** 2015-06-10

**Authors:** Qi Wang, A. Egrinya Eneji, Xiangqiang Kong, Kaiyun Wang, Hezhong Dong

**Affiliations:** 1 Cotton Research Center, Shandong Key Lab for Cotton Culture and Physiology, Shandong Academy of Agricultural Sciences, Jinan 250100, China; 2 College of Plant Protection, Shandong Agricultural University, Tai’an, Shandong 271018, China; National Key Laboratory of Crop Genetic Improvement, CHINA

## Abstract

Many secondary metabolites have insecticidal efficacy against pests and may be affected by abiotic stress. However, little is known of how plants may respond to such stress as pertains the growth and development of pests. The objective of this study was to determine if and how salt stress on cotton plants affects the population dynamics of aphids. The NaCl treatment (50mM, 100mM, 150mM and 200mM) increased contents of gossypol in cotton by 26.8–51.4%, flavonoids by 22.5–37.6% and tannic by 15.1–24.3% at 7–28 d after salt stress. Compared with non-stressed plants, the population of aphids on 150 and 200 mM NaCl stressed plants was reduced by 46.4 and 65.4% at 7d and by 97.3 and 100% at 14 days after infestation. Reductions in aphid population were possibly attributed to the elevated secondary metabolism under salt stress. A total of 796 clones for aphids transcriptome, 412 clones in the positive- library (TEST) and 384 clones in the reverse-library (Ck), were obtained from subtracted cDNA libraries and sequenced. Gene ontology (GO) functional classification and KEGG pathway analysis showed more genes related to fatty acid and lipid biosynthesis, and fewer genes related to carbohydrate metabolism, amino acid metabolism, energy metabolism and cell motility pathways in TEST than in Ck library, which might be the reason of aphids population reduction. A comparative analysis with qRT-PCR indicated high expression of transcripts *CYP6A14*, *CYP6A13*, *CYP303A1*, NADH dehydrogenase and fatty acid synthase in the TEST group. However, *CYP307A1* and two ecdysone-induced protein genes were down regulated. The results indicate that genes of aphids related to growth and development can express at a higher level in reaction to the enhanced secondary metabolism in cotton under salinity stress. The expression of *CYP307A1* was positively correlated with the population dynamics of aphids since it was involved in ecdysone synthesis.

## Introduction

Secondary metabolism is a distinctive process in the growth and development of plant species including cotton, and in their adaptation to the environment [1]. Apart from self-regulations by cotton plant, secondary metabolisms are influenced by biotic and abiotic environmental factors [2,3], and provide considerable defense against pests [4–6]. Sustained drought stress significantly reduced leaf camptothecin (CPT) concentration in *Camptotheca acuminata* seedlings [7], while salinity stress reduced total protein content and increased gossypol contents in cotton tissues [8].

The main secondary metabolites with insecticidal efficacy including gossypol, flavone and tannin can be used in integrated pest management (IPM) [9]. As a polyphenolic compound, (+)—and (-)—gossypol exhibits different levels of biological activities [5] and has biochemical effects on *Helicoverpa armigera* Hübner [10,11]. Increasing CO_2_ levels increased the condensed tannin and gossypol content in cotton plants, and reduced the growth and development of infesting *Aphids gossypii* indirectly [12]. A reduction in the levels of induced terpenoids due to lack of target herbivore induction in Bt cotton increased aphid population [13]. The cotton cultivar, M9101 with high gossypol contents had more antibiotic effect on *A*. *gossypii* and *Bemisia tabaci* than the low-gossypol cultivar, ZMS13 [14,15]. Gossypol, terpenoid aldehydes, hemigossypolone and the heliocides, H1 and H2, are more closely associated with resistance in the tobacco budworm, *Heliothis virescens* [16]. The most prevalent flavonoids, gossypetin 8-0-rhamnoside and gossypetin 8-0-glucoside were also reported to be contributing factors of resistance to tobacco budworm [17]. Compared with non-transgenic cotton, the reductions in gossypol and tannin contents of Bt cotton favored the development of *Tetranychus cinnaberinus* mites [18].

The interaction between plant and insect is multifaceted and complicated. On the one hand, plants produce an array of metabolites that are mainline defenses against pests, e.g., secondary plant compounds in cotton, can enhance the efficacy of Cry toxins and also increase the fitness costs associated with the resistance against the Cry toxins in the target pests [19]; on the other hand, however, herbivorous insects develop a set of effective defense mechanism, including physiological tolerance, behavioral avoidance and enzymatic detoxification systems [20]. Herbivorous insects usually elevate activities of detoxification enzymes, including cytochrome P450 monooxygenases (P450s) to counteract the defensive plant secondary metabolites [21,22]. The CYPs is a large gene family of multifunctional enzymes dealing with the metabolism of insecticides and poisonous secondary metabolites [23,24].

Diversity of P450 substrate recognition sites induces the adaptability of metabolism, and thus multiple P450 genes can metabolize one substrate, or a single P450 gene can metabolize a number of different substrates [25]. Gossypol-induced P450s exhibited high divergence and at least five of them (*CYP321A1*, *CYP9A12*, *CYP9A14*, *CYP6AE11* and *CYP6B7*) contributed to cotton bollworm (*Helicoverpa armigera*) tolerance of deltamethrin [26]. Five genes, *CYP337B1*, *CYP9A12*, *CYP9A14*, *CYP6AE11* and *CYP6B7* showed considerably high expression in the resistant strain of *Helicoverpa armigera* against fenvalerate pesticide [27,28]. The *CYP9A14* and *CYP6AE11* were significantly over-expressed in *Helicoverpa armigera* strains from Burkina Faso and Spain when compared with Heliar, a susceptible strain [29]. The *CYP9A12* and *CYP9A14* had 19- and 4.3-fold overexpression in the midgut of a laboratory-selected strain compared with a field-derived strain of *H*. *armigera* but *CYP6B7* and *CYP4G8* were not over-expressed [30]. These results indicate that gossypol had a significant role in changing bollworm midgut redox state, but bollworm produced resistance to gossypol through the expression of P450. An extensive substrate of P450 genes can administer to the tolerance of insects on more widely toxic secondary metabolites through the ingestion of single or few toxic compounds.

It is still not clear if and how cotton plants under abiotic stress affect the growth and development of pests. The objective of the present study was to determine if cotton under salt stress could decrease the population dynamics of aphids through changing the expression of detoxification and development related genes, such as CYP, fatty acid and lipid biosynthesis. Here we consider the response of aphids at both physiological and transcriptional levels of the secondary metabolism of cotton under salinity stress. By investigating changes in the expression of genes that contribute to aphids detoxification and development, a number of candidate genes related to detoxification were indentified and the expression of many detoxification related genes was examined in Aphids incubated with salt-stressed cotton plants.

## Materials and Methods

### Plant culture and salinity stress

Seeds of SCRC 28 and K638, two commercial Bt (*Bacillus thuringiensis*) transgenic upland cotton (*Gossypium hirsutum* L.) cultivars were sown in plastic boxes (20 cm×15 cm×10 cm) containing wet sand and allowed to germinate and grow under light/dark regimes of 16/8 h and 30°C. When most of the seedlings reached the two true-leaf stage, healthy ones of uniform sizes were selected and transferred to a 1/2 Hoagland’s hydroponic culture. Cotton seedlings with five true leaves were treated with 50, 100, 150 or 200 mM NaCl, while plants irrigated with NaCl-free solution served as the control. The experiment was arranged into a completely randomized design with four replications and 24 plants (8 pots) per replication. The culture solution was renewed every three days.

### Aphid collection and infestation

*Acyrthosiphon gossypii* were collected from hibiscus at Mount Tai (36°22'91.14''N, 117°09'66.67''E), Tai-An, Shandong Province of China in May 2012. The specimen was collected on private land; the owner of the land gave permission to conduct the study on this site. This study did not involve endangered or protected species. A single foundress was used to establish genetically identical individuals in the laboratory. They were incubated with the cotton seedlings in a digital biochemical incubator at 26–28°C, 70–80% RH, under a 16/8h light/dark cycle for one month. Salinity-stressed or non-stressed plants were infested with ten aphids per plant and isolated in net cages. The population of aphids on the terminal buds was counted at a 7d interval after infestation.

Some stressed and non-stressed plants were also infested on the terminal buds with one foundress per plant at 7 d after 100mM NaCl treatment, and aphid oviposition and duration of generation time were examined at 7 d after infestation (DAI).

### Suppression subtractive hybridization and library construction

Aphids reared on SCRC28 cotton plants under 100mM NaCl or non-salinity for two weeks were used for total RNA extraction and transcriptome analysis. About 100 mg of aphids in each treatment were collected into a 1.5 ml centrifuge tube and stored in liquid nitrogen. Total RNAs were extracted using TRIzol reagent (Invitrogen, No.15596-018). Each mRNA sample was purified using FastTrack MAG mRNA isolation Kit (Invitrogen, No. K158002). The cDNA synthesized from salinity groups was used as the tester, and non- salinity groups cDNA as the driver to construct TEST library. On the contrary, the Ck library was constructed using the cDNA from non-salinity groups as tester and the cDNA from salinity groups as the drives. The cDNA samples were digested with RsaI and tester cDNA was separated into two groups and ligated with two different adapters for further hybridization. In the first hybridization, two groups of tester cDNA were hybridized with an excess of driver cDNA at 98°C for 1.5min and 68°C for 8h to enrich the differentially expressed sequences. In the second hybridization, both of the previous reactions were hybridized together in the presence of a fresh driver cDNA at 68°C overnight. After the second hybridization, the subtracted products were amplified by PCR using primers complementary to adapters (applied by the kit). PCR parameters were 94°C for 5min, 27 cycles of 94°C for 30s, 66°C for 30s, and 72°C for 1.5min. A nested PCR reaction of 94°C for 5min, 12 cycles at 94°C for 30s, 68°C for 30s, and 72°C for 1.5min was carried out. The subtracted PCR products were cloned into the pMD18-T Simple Vector (Invitrogen, Shanghai,China), and transformed into DH5α strain (Invitrogen, Shanghai, China). The white colonies were checked by PCR with vector specific primers and electrophoresed on 1% agarose gel and sequenced using ABI PRISM Big Dye Terminator Cycle sequencing Core kit with AmpliTaq DNA polymerase (ABI, Foster City, CA), performed by Invitrogen Biotech. The sequence results were compared with the protein database using BLAST [31] at the National Center for Biotechnology Information (NCBI), Betheesda, Maryland, USA.

### Quantitative Real-Time PCR

qRT-PCR was used to determine the transcript levels of some important genes including P450, NADH, lipid biosynthesis, juvenile hormone binding protein (Jhbp) and ecdyson. About 50 mg of aphids which reared on SCRC28 cotton plants for two weeks were collected and then extracted for total RNA using TRIzol reagent (Invitrogen, No.15596-018). Aphids reared on non- and salt-treated SCRC28 cotton plants were denoted as NS and ST, respectively. The first-strand cDNA was synthesized from 1μg of total RNA with First-Strand Synthesis SuperMix for qRT-PCR kit (Invitrogen). qRT-PCR reactions were performed using the iCycler iQ2 sequence detection system (Bio-Rad, Hercules, CA) with SYBR Green PCR Master Mix (Applied Biosystems). The specific primers are listed in Table 1. The PCR parameters were 95°C for 3min; 40 cycles at 95°C for 10s, at annealing temperatures of 60°C for 10s, and 72°C for 10s. Three independent biological replicates of each treatment were performed and normalized to β-actin expression.

**Table 1:** Sequence of primers used in qRT-PCR.

Gene	Forward primers	Reverse primers
Actin	CTGGTATGTGCAAAGCCGG	GTCTTTTTGTCCCATACCGACC
CYP6A14	ATTCGCTGAAAGACTCGC	AAGAACACTCGGACCATT
CYP6A13	ATTCAAGACAGAGTACGCAAAG	AAAGCAACCAGTGGAGGA
CYP307A1	CATTGCCGTTCGTTACTT	AGGACCACCTGCGTTTCA
CYP6A2	AGAGCCAAGTTGAGTCCG	TTTCGTTATCTGCCCATT
CYP303A1	TCCTCAAAGCAGAACTCG	CAGATCCAGCCATAAACATA
NADH 1	AGAAACCGTAGCAGTAGAAGA	CATTGGTCAGCACTTGGT
NADH 2	GCAACCCTCAAACCACAA	TACAAGCGTAGCCTCCATC
Fatty acid synthase 1	AATCGGCACATTTGACCT	TCCTTGCTAACGCCTTCC
Fatty acid synthase 2	AACCCCAACTGGTCCTAA	AATCGTCCAGCAATACCC
Jhbp 1	ATCCCCTCCAACTTACTG	CGACAAGCCATCTCCTAT
Jhbp 2	ACAAGACACGGTTACGCT	GTGCCCTATGACAGTTGG
Ecdysone1	CATTCTCCCTGCCCTACA	TCCATCGAACACTCCTCA
Ecdysone2	CCGGATAGCGAGGAACAGC	CGTAGAAGGTGAGCGAAAG

### Determination of secondary metabolites

Secondary metabolites in plant tissues were determined at a 7-d interval after salinity treatment. Apical points of the main stem together with the 1st true leaf from the main-stem terminal were sampled and quickly frozen in liquid nitrogen, then freeze-dried at -80°C, grounded into powder, and stored at -20°C in a freezer.

The contents of total flavonoids were determined using the Al(NO_3_)_3_-NaNO_2_, chromogenic system [32]; the contents of gossypol were determined according to Smith(1967)using ultraviolet spectrophotometer [33]; and those of tannin according to Folin’s assay [34].

### Statistical analysis

The data for aphid population and allelochemical contents were analyzed using ANOVA in the SPSS system followed by a R-E-G-W F(R) multiple comparison test at *P*<0.05. The data for transcript levels were analyzed by t-test with a cutoff value of FDR<0.05.

On the basis of NCBI Nt annotation, the WEGO software was used to perform GO functional classification for TEST and Ck library unigenes [35]. The Cluster of Orthologous Groups (COG) is a database where the orthologous gene products were classified. All unigenes were aligned to the COG database to predict and classify possible functions [36].

## Results

### Population dynamics of aphids

**Fig 1:**
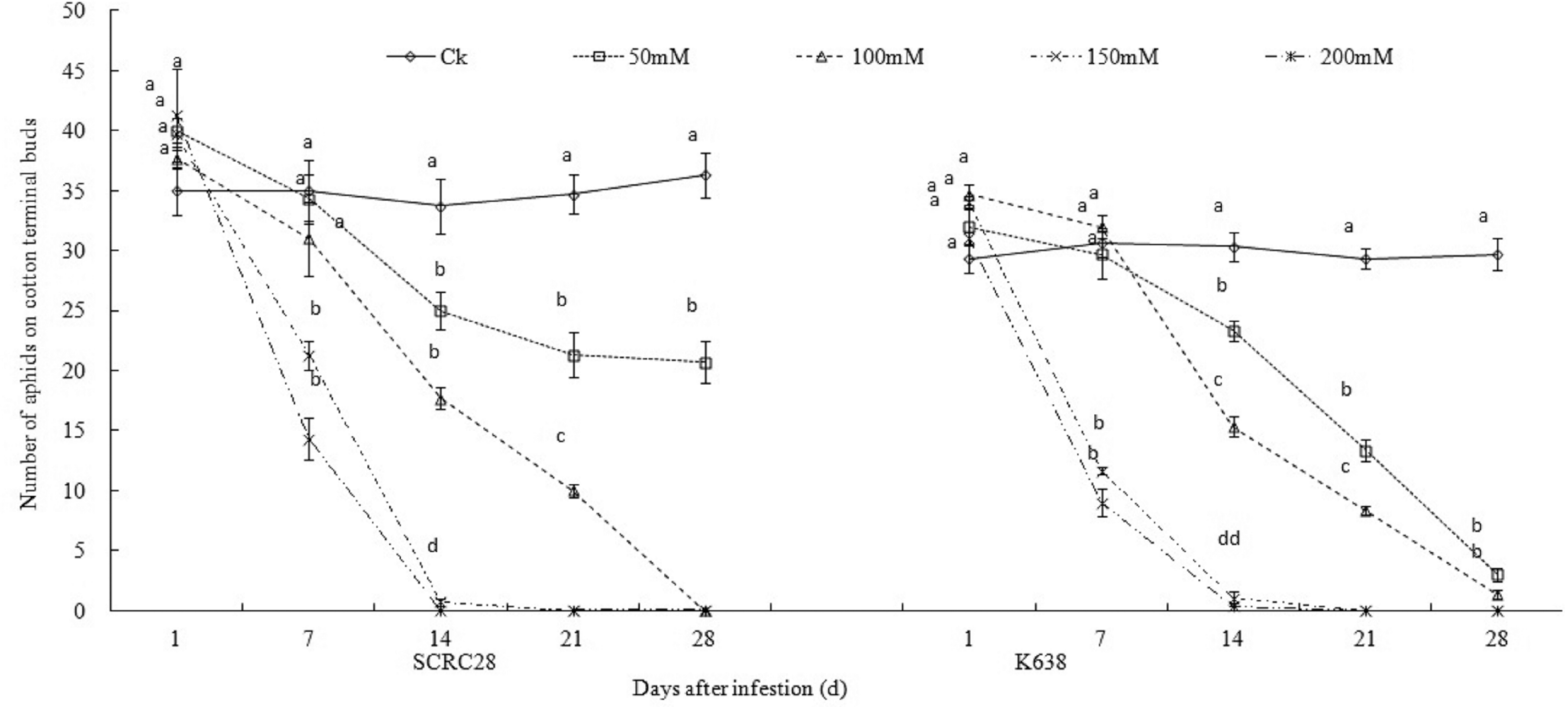
Population dynamics of aphids on the cotton cultivars, SCRC28 and K638 under NaCl stress. Observation was started from aphid infestation. Error bars represent ±SD. Bars bearing different letters are significantly different at *P*<0.05.

At 7 days after infestation (DAI), the occurrence of aphids on SCRC 28 under 50 and 100 mM NaCl treatments was roughly the same as that under non-stressed control, but the occurrence decreased by 46.3% under 150 mM NaCl and 65.4% under 200 mM NaCl. At 14 DAI, the occurrence of aphids on SCRC 28 decreased by 37.5, 53.1, 98.2 and 100% under 50, 100, 150 and 200 mM NaCl, respectively (Fig 1). Similar reductions were obtained for K638. The results showed that salt-stressed cotton could significantly reduce the occurrence of aphids; the higher the salinity, the smaller the occurrence (Fig 1).

### Reproductive performance of aphids

Salt–stressed cotton significantly reduced the generation time and oviposition of the infesting aphids. Averaged for the two cotton cultivars, the 50, 100, 150, and 200 mM NaCl treatments reduced the generation time by 16.0, 24.0, 27.0 and 28.6%, and oviposition by 23.8, 27.0, 36.5 and 47.6%, respectively (Table 2).

**Table 2:** Generation time and oviposition of aphids infesting cotton under different NaCl stress.

Cultivars	NaCl concentration (mM)	Generation time(day)	Oviposition
SCRC28	0	5.5±0.3a	6.3±0.3a*
	50	6.4±0.1b	5.0±0.0b
	100	6.8±0.1b	4.6±0.3b
	150	7.0±0.1b	4.0±0.0c
	200	6.7±0.1b	3.0±0.3c
K638	0	4.5±0.3a*	6.3±0.3a
	50	5.2±0.2b	4.6±0.3b
	100	5.6±0.1c	4.6±0.3b
	150	5.7±0.1c	4.0±0.0b
	200	6.1±0.2d	3.6±0.3b

* Values (Mean ± SD) followed by different letters indicates significant difference at *P* <0.05 according to Fish’s test.

### Contents of secondary metabolites

**Fig 2:**
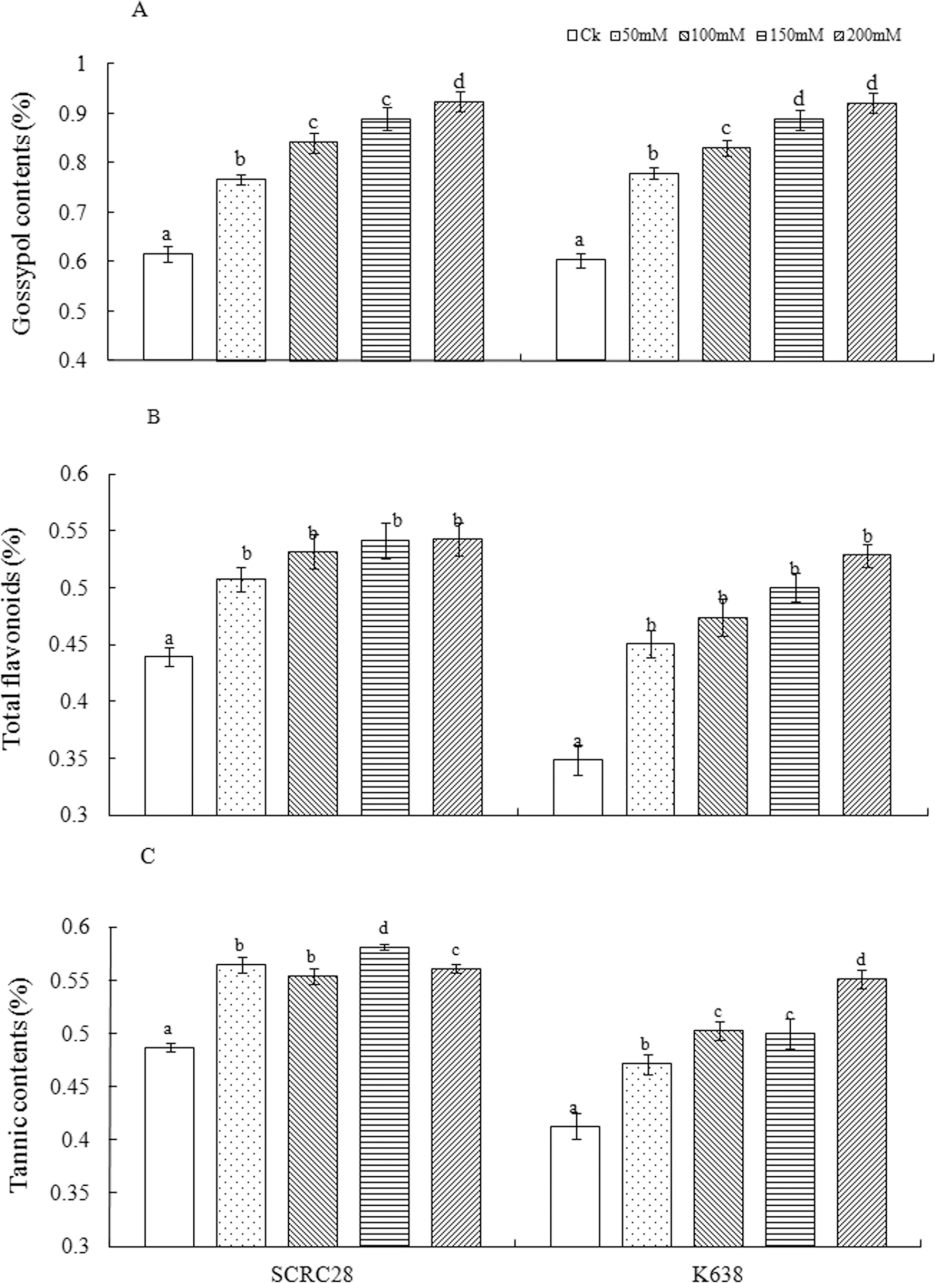
The effect of different NaCl stress on secondary metabolites of two cotton cultivars at 7 days after infestation (DAI). (A) Gossypol contents of SCRC28 and K638; (B) Total flavonoids contents of SCRC28 and K638; (C) The Tannin contents of SCRC28and K638. For each treatment, error bars represent standard deviation. Bars bearing different letters are significantly different at *P*<0.05.

Salinity stress significantly affected the accumulation of secondary metabolites in plant tissues. Averaged across the two cultivars at 7 days after salt stress, the 50, 100, 150 and 200 mM NaCl treatments increased the contents of gossypol by 26.8, 37.1, 45.8 and 51.4% (Fig 2A), those of flavonoids by 22.5, 28.6, 33.4 and 37.6% (Fig 2B), and those of tannin by 15.1, 17.7, 20.2 and 24.3% (Fig 2C), respectively. The results indicated that salinity stress enhanced the secondary metabolism as indicated by the increased accumulation of gossypol, flavonoids and tannin.

### Subtracted cDNA library from aphids

A total of 796 clones were obtained and sequenced from the subtracted cDNA libraries, including 412 clones in the positive-library (TEST) and 384 clones in the reverse-library (Ck), which effectively enriched the differentially expressed genes with the recombination rate up to 95%. The average sizes of insert fragments were 222 and 313 bp respectively. The BLAST results of the 796 sequenced clones showed similarity with *Acyrthosiphon pisum* genes in the GenBank database. The Unigene annotion statistics is summarized in Table 3.

**Table 3:** Unigenes annotion statistics from the subtracted cDNA library.

Database	TEST libary			Ck libary		
	No.	%	E cutoff	No.	%	E cutoff
Nt	378/412	90.8	1e-5	345/384	89.8	1e-5
Nr	289/412	70.1	1e-5	281/384	73.2	1e-5
Swissprot	115/412	27.9	1e-10	160/384	41.7	1e-10
COG	50/412	12.1	1e-10	94/384	24.5	1e-10
Kegg	229/412	55.6	1e-10	246/384	64.1	1e-10
Interpro	114/412	27.7	InterProScan 5	170/384	44.3	InterProScan 5
GO	86/412	20.9	InterProScan 5	139/384	63.2	InterProScan 5

**Fig 3:**
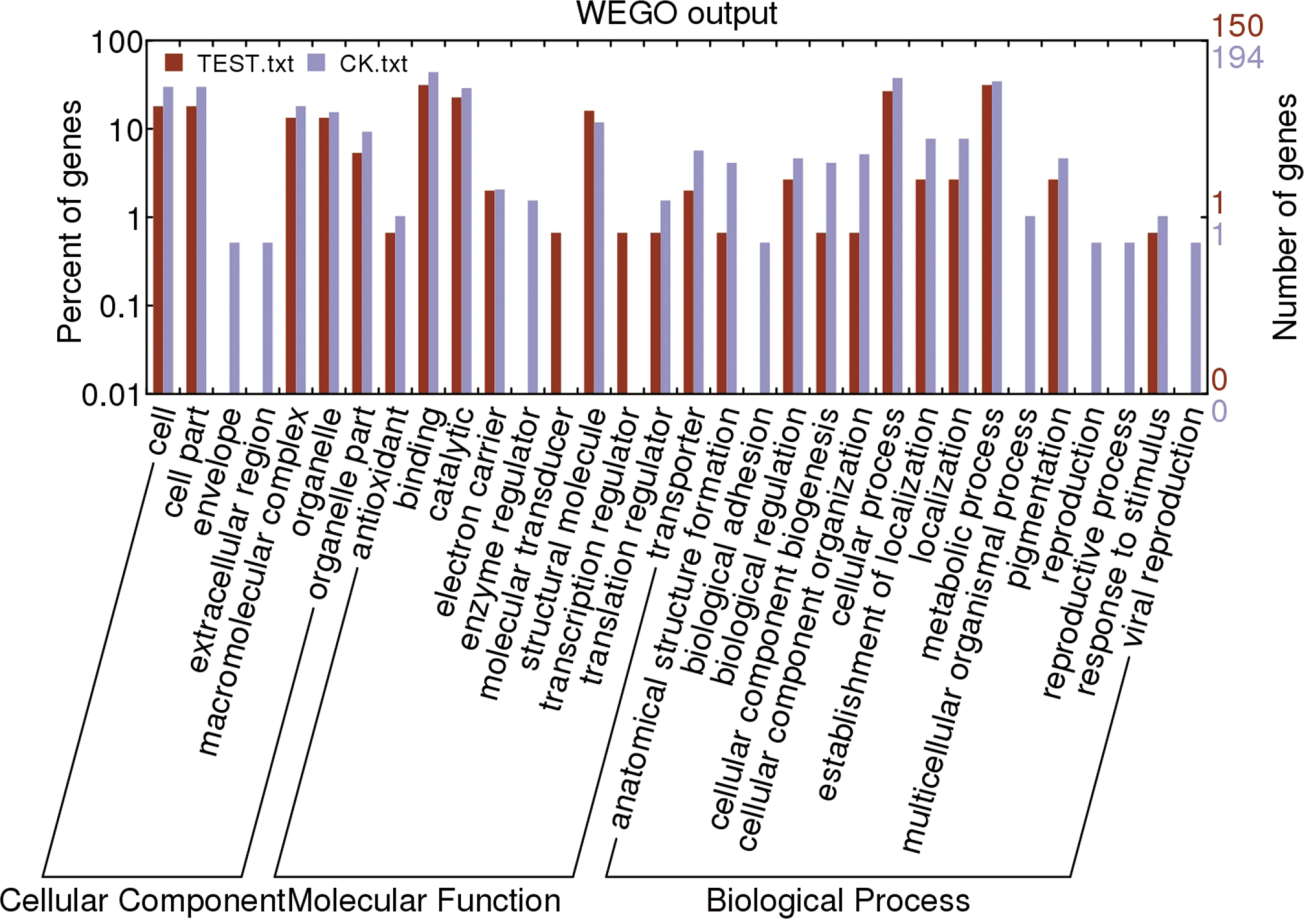
Histogram of Gene Ontology classification from the subtracted cDNA libraries. The results are summarized in three main categories: biological process, cellular component and molecular function.

**Fig 4:**
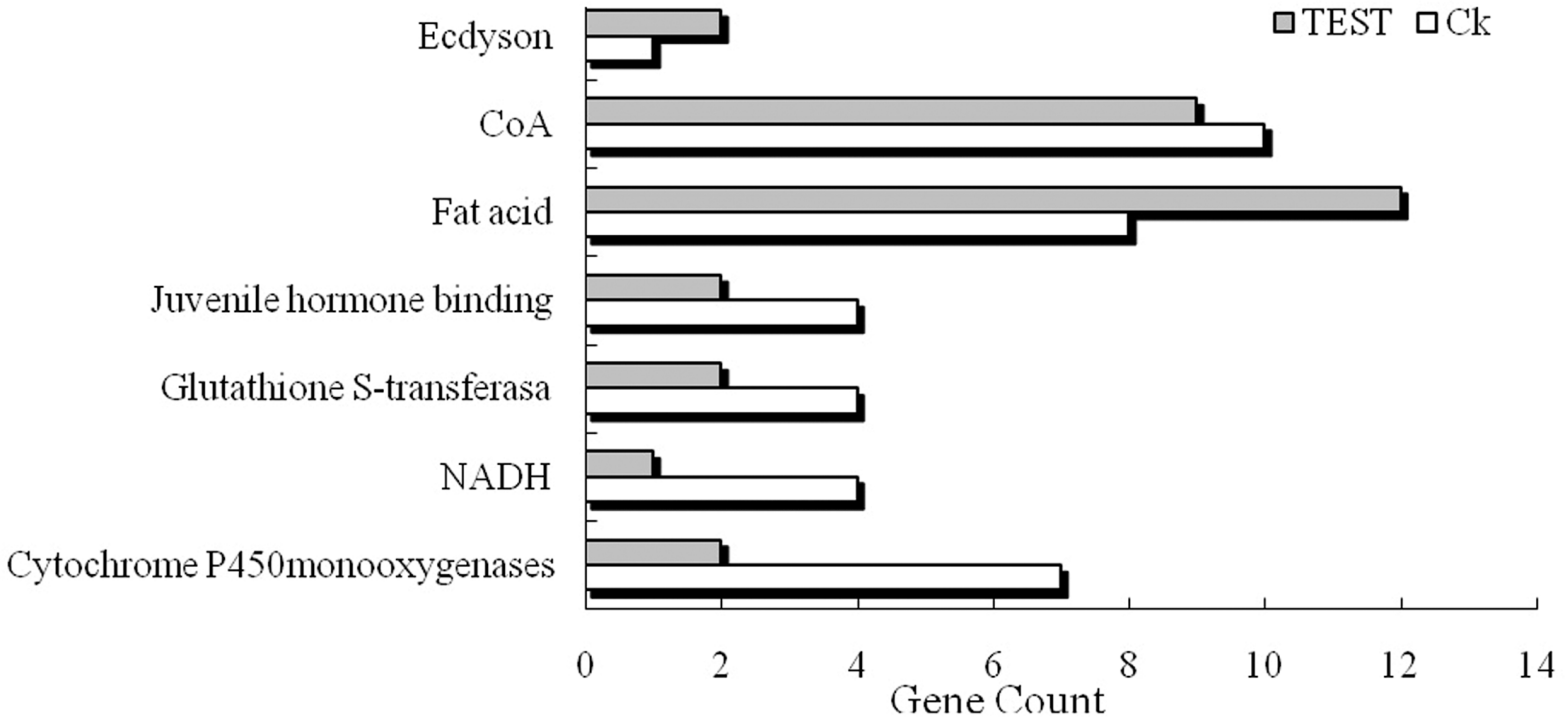
Differentially expressed genes enriched 7 specific Gene Ontology terms. The abscissa of the bar plot represents the gene count within each GO category. All processes listed had enrichment p values<0.05.

Gene ontology (GO) analysis was performed by mapping differentially expressed genes into the records of the GO database (http://wego.genomics.org.cn/cgi-bin/wego/index.pl), 86 transcripts in TEST library and 139 transcripts in Ck library were annotated with GO terms and can be classified into 24 and 30 secondary level categories, respectively, including two secondary categories of molecular function category (molecular transducer activity and transcription regulator activity) only displayed in TEST library (Fig 3 and S1 and S2 Texts). The GO annotation of seven enriched genes is presented in Fig 4. Fatty acid and CoA associated to lipid metabolism were the most frequently expressed sequence tag (EST), with 21 clones in TEST library and 18 clones in Ck library. Fifteen detoxifying enzyme genes mentioned to Cytochrome P450 monooxygenase and glutathione S transferase (GST) were also found in the libraries with 11 clones of them in Ck library and the rest in TEST library. Six juvenile hormone binding and 3 ecdyson related genes were found in the libraries, with more juvenile hormone binding but fewer ecdyson genes in the Ck than TEST library. As for enzyme category, there were four clones of NADH dehydrogenase in Ck library and only one clone in TEST library.

To understand the functions of the differentially expressed genes, we mapped all the genes to terms in KEGG database to searching for genes that were significantly enriched (Table 4). Among the differentially expressed genes with KEGG pathways annotation, there were six genes and two genes in lipid metabolism pathway which associate to fatty acid and lipid biosynthesis, in TEST and Ck library, respectively. Notably, more genes associated to carbohydrate metabolism, amino acid metabolism, energy metabolism and cell motility pathways were found in Ck than TEST library (Table 4). Two and one genes associated to signal transduction and biosynthesis of secondary metabolites were found in TEST library but not in Ck library (Table 4).

**Table 4:** KEGG pathway annotation of differentially expressed genes obtained from the subtracted cDNA library.

Pathways	Ck (gene No.)	TEST(gene No.)
Carbohydrate Metabolism; Citrate cycle (TCA cycle)	6	1
Energy Metabolism; Oxidative phosphorylation	12	5
Glycan Biosynthesis and Metabolism; Proteoglycans	0	1
Lipid Metabolism; Fatty acid biosynthesis	1	3
Lipid Metabolism; Lipid biosynthesis proteins	1	3
Metabolism of Other Amino Acids; Glutathione metabolism	4	1
Amino Acid Metabolism; Alanine, aspartate and glutamate metabolism	4	2
Amino Acid Metabolism; Arginine and proline metabolism	2	1
Carbohydrate Metabolism; Inositol phosphate metabolism	0	1
Signal Transduction; Phosphatidylinositol signaling system	0	2
Carbohydrate Metabolism; Glycolysis / Gluconeogenesis	3	0
Carbohydrate Metabolism; Glyoxylate and dicarboxylate metabolism	1	0
Cell Motility; Cytoskeleton proteins	23	5
Biosynthesis of Other Secondary Metabolites;Isoquinoline alkaloid biosynthesis	0	1

### Analysis of related gene expression with qRT-PCR

**Fig 5:**
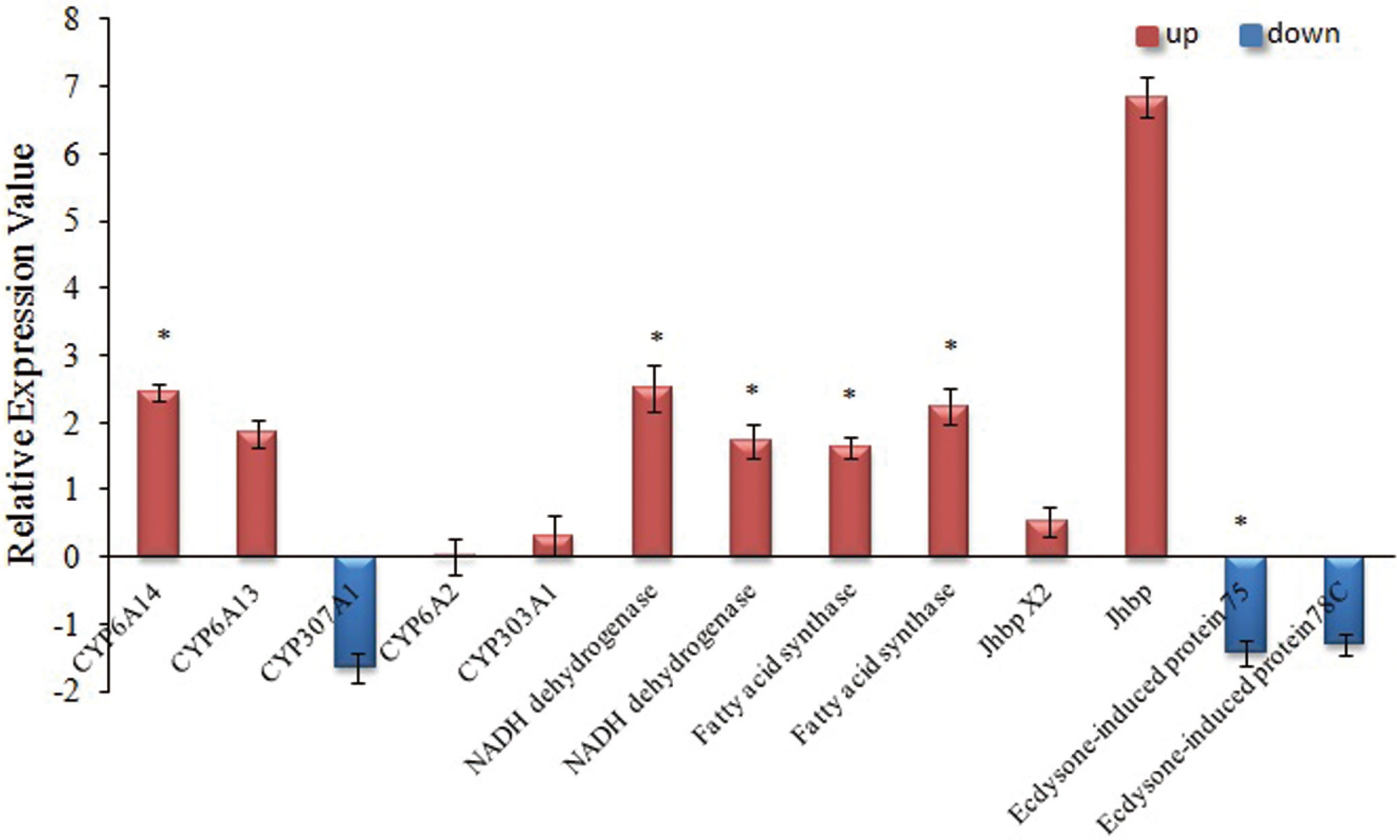
The RT-PCR analysis of genes involved in Cytochrome P450 and Aphids growth. The x-axis indicates thirteen different genes. The y-axis indicates the relative expression value of uingene. Bars bearing asterisk are significantly different at *P*<0.05.

To further test the components of the subtracted cDNA library, qRT-PCR analysis was performed with specific primers for a subset of fourteen genes, which have been identified by SSH. The results showed that five significantly up-regulated genes were found in TEST library and one significantly down-regulated genes were found in Ck library, indicating that the SSH library was validated (S1 Table). The transcript levels of the three P450 genes (*CYP6A14*, *CYP6A13*, *CYP303A1*) in aphids reared on salt- treated cotton (ST) were higher than in non-salt treated (NS); among these, the expression level of *CYP6A14* genes of ST aphids was 2.46 folds higher than NS (S1 Table). In contrast, *CYP307A1* showed down regulation when reared on ST (log_2_FC = -1.65). The expression levels of all the NADH dehydrogenase, lipid biosynthesis and juvenile hormone binding genes of aphids reared on ST were all higher than NS aphids, but the expression levels of two ecdysone-induced protein genes were lower than NS aphids (Fig 5 and S1 Table).

## Discussion

Secondary metabolites are adaptive traits which have been subjected to the variable nature during evolution [37]. Proper use of secondary metabolic allelochemicals in plants is an important approach to controlling herbivorous insects in integrated pest management [4,38]. Our findings indicate that the reduced severity of aphid infestation in cotton plants might be attributed to the enhanced level of secondary metabolites as indicated with increased levels of gossypol, flavonoids and tannin in cotton tissues under salt stress. It has been reported that a high diversity of secondary metabolite mixture in high concentration provides a more effective protection against herbivores than a single compound or a low diversity mixture in both low and high concentrations [39]. Our data support the hypothesis about the function of diverse plant secondary metabolites. Mixed concentrations of gossypol, flavonoids and tannin in plant tissues acting against aphid population ranged from 0.47 to 0.92% under salinity. High concentrations of allelochemicals resulted in low oviposition of aphids, but a relatively low concentration of the three compound mixtures also resulted in low oviposition, suggesting that a moderate diversity or synergistic reaction may be equally functional in low and high concentrations. The aphids was not affected by metabolite concentrations of the cotton, however, an increasing content of allelochemicals in the cotton reduced the aphid population slightly in low NaCl (50–100 mM) but considerably in high NaCl (150–200 mM). The cotton secondary metabolites seemed to be equally as effective in reducing oviposition as botanical insecticides like rotenone (Rot), azadirachtin (Aza) and paeonolum (Pae) [40].

In response to the toxic secondary metabolites in plants, insects usually increase interrelated P450 expression to adjust their defense [25]. Arrays of P450s have been focused on insect tolerance of insecticide and toxic allelochemicals. *Drosophila CYP307A1* is involved in ecdysone synthesis, of which the expression level positively correlates with the hemolymph ecdysteroid titer [41]. *Drosophila melanogaster CYP307A1* is the ancestral gene and the closest ortholog of the coleopteran, lepidopteran and mosquito *CYP307A* subfamily genes. The duplications of ancestral P450 genes that acquired novel functions might be an important mechanism for evolving the ecdysteroidogenic pathway [42]. In this study, the expression level of *CYP307A1* positively correlated with two ecdysone-induced protein genes under salt stress. The results also suggest that *Drosophila CYP307A1* may be involved in ecdysone synthesis.

It has been reported that the *CYP6A* gene of housefly, *Musca domestica* can regulate the metabolism of plant terpenoids [43] and the honey bee, *Apis mellifera* CYP6AS enzymes can also metabolize flavonoids and quercetin [24,44], and *CYP6CX1* were associated with thiamethoxam resistance in *Bemisia tabaci* [45] *Plutella xylostella* can employ cytochrome P450, glutathione *S*-transferase (GST), chemosensory protein (CSP) and other important protein to defense the toxin-rich environment [46].

## Conclusions

Genes responsible for the growth and development of aphids expressed at a higher level against the enhanced secondary metabolism under salinity stress. More genes related to fatty acid and lipid biosynthesis, but fewer genes related to carbohydrate metabolism, amino acid metabolism, energy metabolism and cell motility pathways were found from subtracted cDNA library, suggesting that decreased carbon, nitrogen and energy metabolism of aphids reared on salt-treated cotton were attributed to aphids population reduction under salt stress. *CYP307A1* was involved in ecdysone synthesis and thus be positively correlated with the population dynamics of aphids. The interaction between plant secondary metabolism and regulation of resistance by herbivores cyptochrome P450 genes directly stimulated cotton secondary metabolism, but also indirectly affected the growth and development of aphids in their tolerance of toxic allelochemicals. These results should aid the development of an integrated pest management for herbivorous insects infesting cotton grown under saline conditions.

## Supporting Information

S1 Text The Gene ontology (GO) database of the positive-library (TEST) in this study. (TXT)

S2 Text The Gene ontology (GO) database of the reverse-library (Ck) in this study. (TXT)

S1 Table Differentially expressed genes in aphids TEST and Ck groups. (DOCX)
