# Supplementary material for: Salt Stress Effects on Secondary Metabolites of Cotton in Relation to Gene Expression Responsible for Aphid Development
Source: PLoS One. 2015 Jun 10;10(6):e0129541. doi: 10.1371/journal.pone.0129541 (PMC4489590; doi:10.1371/journal.pone.0129541)
Supplement: S1 Table — (DOCX) [file pone.0129541.s003.docx]

S1 Table. Differentially expressed genes in aphids TEST and Ck groups.

| Gene | log_2_FC(TEST/Ck) | Regulation | FDR | Significant | Transcript_ID |
| --- | --- | --- | --- | --- | --- |
| *CYP6A14* | 2.46 | up | 0.0049 | yes | ACYPI064758 |
| *CYP6A13* | 1.84 | up | 1.0000 | No | ACYPI000619 |
| *CYP307A1* | -1.65 | down | 1.0000 | No | ACYPI001519 |
| *CYP6A2* | 0.01 | up | 0.9925 | No | ACYPI000990 |
| *CYP303A1* | 0.31 | up | 0.8577 | No | ACYPI003371 |
| NADH dehydrogenase | 2.52 | up | 0.0461 | yes | ACYPI008219 |
| NADH dehydrogenase | 1.72 | up | 0.0093 | yes | ACYPI31567 |
| Fatty acid synthase | 1.63 | up | 0.04719 | yes | ACYPI007475 |
| Fatty acid synthase | 2.24 | up | 0.0031 | yes | ACYPI008540 |
| Jhbp X2 | 0.52 | up | 0.6724 | No | ACYPI006428 |
| Jhbp | 6.84 | up | 0.5268 | no | ACYPI084038 |
| Ecdysone-induced protein 75 | -1.44 | down | 0.0390 | yes | ACYPI007773 |
| Ecdysone-induced protein 78C | -1.30 | down | 0.1472 | no | ACYPI002307 |
